# *Giardia duodenalis* in patients with diarrhea and various animals in northeastern China: prevalence and multilocus genetic characterization

**DOI:** 10.1186/s13071-022-05269-9

**Published:** 2022-05-11

**Authors:** Yanchen Wu, Lan Yao, Hongshuang Chen, Weizhe Zhang, Yanyan Jiang, Fengkun Yang, Aiqin Liu, Yujuan Shen

**Affiliations:** 1 Department of Parasitology, Harbin Medical University, Harbin, 150081 Heilongjiang China; 2 National Institute of Parasitic Diseases, Chinese Center for Disease Control and Prevention (Chinese Center for Tropical Diseases Research), NHC Key Laboratory of Parasite and Vector Biology, WHO Collaborating Centre for Tropical Diseases, National Center for International Research on Tropical Diseases, Shanghai, 200025 China

**Keywords:** *Giardia duodenalis*, Multilocus genotyping, Assemblage, Sub-assemblage, Diarrheal patients, Mammals, Zoonotic

## Abstract

**Background:**

*Giardia duodenalis* is a common parasitic diarrheal agent in humans, especially in developing countries. The aim of this study was to investigate the prevalence and multilocus genetic characterization of *G. duodenalis* in patients with diarrhea and animals in northeastern China, and to assess the epidemiological role of animals in the transmission of human giardiasis.

**Methods:**

A total of 1739 fecal specimens from 413 diarrheal patients and 1326 animals comprising 16 mammal species were collected in Heilongjiang Province of China and screened for *G. duodenalis* by PCR and sequencing of the *SSU rRNA* gene. All *G. duodenalis*-positive specimens were subtyped by PCR and sequencing of the *bg*, *tpi*, and *gdh* genes. To detect additional mixed infections of different assemblages, assemblage A/B/E-specific PCRs were performed to amplify the *tpi* gene.

**Results:**

Sequence analysis of the *SSU rRNA* gene determined the prevalence of *G. duodenalis* (5.81%, 24/413) in diarrheal patients, with a peak in minors aged 5–17 years, and identified assemblages A and B. MLG-AII and MLG-B1 were obtained based on concatenated nucleotide sequences of the *bg*, *tpi*, and *gdh* genes, with MLG-AII being identical to a cat-derived isolate reported previously. By sequence analysis of the *SSU rRNA* gene, *G. duodenalis* was detected in 214 (16.14%) animals belonging to 11 mammal species, with the prevalence ranging from 1.69 to 53.85%, and assemblages A to G were identified. Sequence analysis of the *bg*, *tpi*, and *gdh* genes from 46 specimens produced 31 MLGs, including MLG-AI (*n* = 1), MLG-B2–B8 (*n* = 18), and MLG-E1–E23 (*n* = 27).

**Conclusions:**

The finding of *G. duodenalis* in diarrheal patients enhances consciousness of detecting *G. duodenalis* in clinical practice and emphasizes the importance of health education in local inhabitants, especially in the age group of 5–17 years. The identification of seven assemblages (A to G) and 33 MLGs reveals genetic heterogeneity of *G. duodenalis* in the investigated areas. Due to insufficient homology data on the zoonotic transmission of *G. duodenalis*, the precise epidemiological role that animals play in the transmission of human giardiasis needs to be assessed by more large-scale molecular epidemiological investigations of local humans and animals.

**Graphical Abstract:**

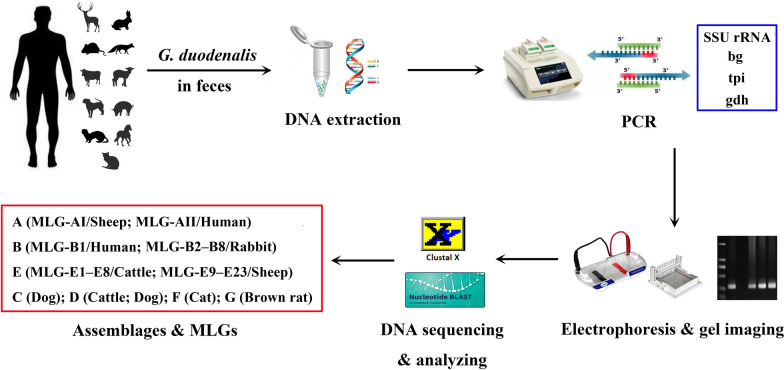

**Supplementary Information:**

The online version contains supplementary material available at 10.1186/s13071-022-05269-9.

## Background

Diarrhea is a common disease in developing countries and is associated with high rates of mortality among young children, with children constituting 1.9 million of the 2.2 million deaths every year [1]. The World Health Organization (WHO) reports diarrheal diseases as one of the top 10 global causes of death [2]. The most common cause of diarrhea is an infection of the intestines due to viruses, bacteria, or parasites. However, parasites do not receive enough attention, especially protozoa such as *Giardia duodenalis* (also known as *G. lamblia* or *G. intestinalis*). In fact, giardiasis has been included in WHO’s Neglected Diseases Initiative since 2004 [3]. *Giardia duodenalis* is a widespread intestinal protozoan in humans, with the prevalence being approximately 10% [4]. The spectrum of clinical manifestations of *G. duodenalis* infection is quite variable, ranging from asymptomatic infection to acute or chronic diarrhea as well as other symptoms of the digestive system (abdominal pain, nausea, flatulence, vomiting, bloating) [5]. *Giardia duodenalis* can lead to growth and developmental retardation in children, even in asymptomatic cases [6]. The evolution and severity of *G. duodenalis* infection greatly depend on the interaction between the host factors (such as age and health status) and the parasite factors (such as the virulence of the parasite and the number of cysts in hosts) [7]. Potentially life-threatening protracted diarrhea may occur in immunocompromised or immunosuppressed individuals, such as those with acquired immunodeficiency syndrome (AIDS), malnourished children, and the elderly [1]. As an important zoonotic intestinal protozoan, *G. duodenalis* has also been detected in more than 40 animal species [8], with prevalence ranging from 1.09 to 100% [9]. Transmission of *G. duodenalis* is mainly through the fecal–oral route via consumption of food or water contaminated by cysts as well as direct contact with infected humans or animals [5].

*Giardia duodenalis* is a complex species, consisting of eight assemblages A to H with different host specificity. Among them, assemblages A and B have the ability to infect humans and a variety of mammals, displaying zoonotic potential for their wide host range. In contrast, assemblages C to H are host-specific for nonhuman species (canids, domestic mammals, cats, rodents, and pinnipeds) [8]. However, assemblages C to F are also occasionally identified in humans, such as assemblage C in China and Slovakia [10, 11], assemblage D in Germany [12], assemblage E in Egypt, Brazil, and Australia [13–20], and assemblage F in Slovakia [21, 22]. Assemblage A is divided into three sub-assemblages (AI, AII, and AIII); however, there is no recognized nomenclature for assemblages B to H [23]. Currently, the most commonly used loci for typing *G. duodenalis* are the small subunit ribosomal RNA (*SSU rRNA*), structural gene (β-giardin [*bg*]), and housekeeping genes (triosephosphate isomerase [*tpi*] and glutamate dehydrogenase [*gdh*]) [24]. Based on difference in the rates of nucleotide substitution within genes, the conserved *SSU rRNA* gene is traditionally used for species and assemblage differentiation, whereas the other three variable genes (*bg*, *tpi*, and *gdh*) are used for both genotyping and subtyping [23]. To date, studies have largely relied on genetic characterization of *G. duodenalis* at one or two genetic loci to determine the prevalence and assess possible zoonotic transmission. Genotyping data and genetic information based on a single locus frequently do not detect mixed infections, and are not sufficient to provide an accurate assessment of possible zoonotic transmission. High-resolution multilocus genotyping tools have been increasingly used to characterize *G. duodenalis* infection in humans and animals. They not only allow the identification of mixed infections of different assemblages in the same specimens, but also enable a better understanding of the zoonotic potential of human-pathogenic assemblages, especially for assemblages A and B [25].

In China, epidemiological investigations and case reports on *G. duodenalis* infection in humans are common, with approximately 28.5 million cases of *G. duodenalis* infection estimated in 2005 [26]. Since genotyping and subtyping tools were used in the identification of human cases of giardiasis in 2000, molecular epidemiological investigations of human *G. duodenalis* infection have been carried out in five provinces/municipalities, with three assemblages (A, B, and C) being identified [25, 27, 28]. However, the prevalence of *G. duodenalis* in diarrheal patients in China is unclear. In northeastern China’s Heilongjiang Province, *G. duodenalis* infection has ever been documented in humans in about 70% of counties/cities based on the first national epidemiological investigation conducted in 1988–1992 [29]. Since then, there have been no reports of *G. duodenalis* infection in humans. To investigate the prevalence and multilocus genetic characterization of *G. duodenalis* in patients with diarrhea and in animals, and to assess the epidemiological role of animals in the transmission of human giardiasis, a molecular epidemiological study of *G. duodenalis* was carried out in patients with diarrhea and various animals by multilocus genotyping of *G. duodenalis* isolates.

## Methods

### Sources and collection of fecal specimens

**Fig. 1:**
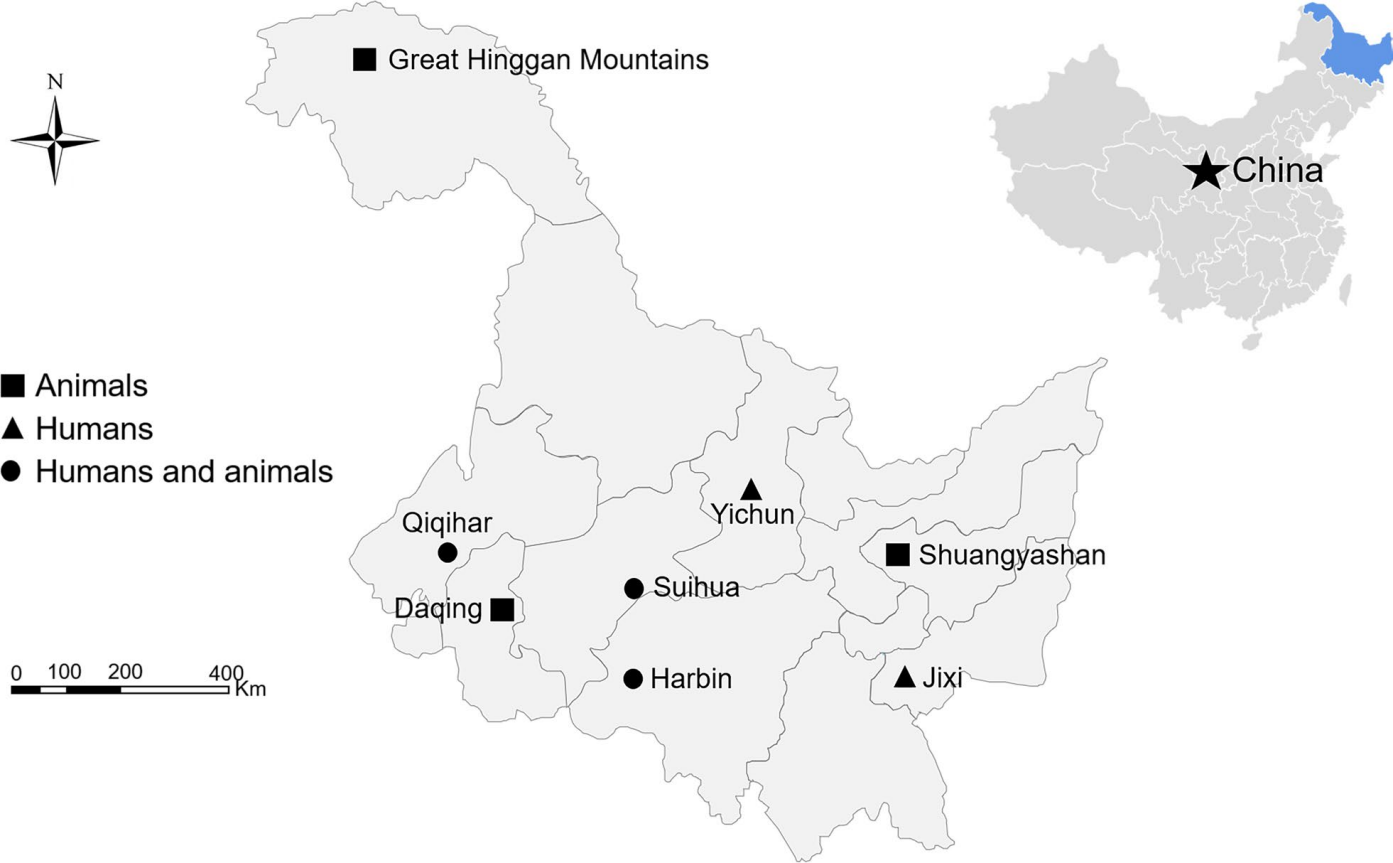
Locations of human and animal fecal specimen collection in this study

A total of 1739 fecal specimens (approximately 5–10 g) were collected from humans and animals (one each) from October 2010 to May 2021 in Heilongjiang Province, China. Of these, 413 human fecal specimens were from patients with diarrhea at least three times each day and who were outpatients in five hospitals: the Second Affiliated Hospital of Harbin Medical University in Harbin (*n* = 150), Central Hospital of Yichun Forestry Administration Bureau in Yichun (*n* = 222), Jianhua Hospital in Qiqihar (*n* = 26), Jidong Central Hospital in Jixi (*n* = 8), and Kangying Hospital in Suihua (*n* = 7) (Fig. 1). They comprised 26 children (< 5 years), 225 minors (5–17 years), 31 young adults (18–35 years), 83 middle-aged adults (36–60 years), and 48 older adults (> 60 years) (Table 1). The remaining 1326 animal fecal specimens were from 16 mammal species, distributed in six cities/areas: Harbin (*n* = 682), Suihua (*n* = 366), Shuangyashan (*n* = 22), Qiqihar (*n* = 31), Daqing (*n* = 18) and the Greater Khingan Mountains (*n* = 207) (Table 2, Fig. 1). None of the animals had any apparent clinical symptoms of diarrhea at the time of sampling. Collected fecal specimens were kept in cool boxes with ice packs, and transported to our laboratory within 48 h. They were stored in a refrigerator at 4 °C (≤ 2 days) or −20 °C (> 2 days) prior to use in the subsequent molecular analysis.

**Table 1:** Prevalence, assemblages, sub-assemblages, and MLGs of *G. duodenalis* in humans in Heilongjiang Province

Age group (years)	Positive no./examined no. (%)	Assemblage (*n*)	Sub-assemblage (*n*)/accession no.			MLGs (*n*)
		*SSU rRNA*	*bg*	*tpi*	*gdh*	
Children (< 5)	0/26					
Minors (5–17)^a^	22/225 (9.78)	A (3)	AII (1)/OM001823	AII (1)/OM001842	AII (1)/OM001857	MLG-AII (1)
		B (19)			**B1 (1)/OM001858**	
			B1 (1)/OM001824	B1 (1)/OM001843	B2 (1)/OM001859	MLG-B1 (1)
Young adults (18–35)	0/31					
Middle-aged adults (36–60)^a^	1/83 (1.20)	A (1)	AII (1)/OM001823			
Older adults (> 60)	1/48 (2.08)	A (1)				
Total	24/413 (5.81)	A (5), B (19)	AII (2), B1 (1)	AII (1), B1 (1)	AII (1), B1 (1), B2 (1)	

One novel sequence obtained in the present study is highlighted in bold

^a^A significant difference in prevalence is observed only between the age groups of 5–17 and 36–60 years

**Table 2:** Prevalence, assemblages, sub-assemblages and MLGs of *G. duodenalis* in different animals in Heilongjiang Province

Host^a^	Positive no./examined no. (%)	Assemblage (*n*)	Sub-assemblage (*n*)/accession no.			MLGs (*n*)
		*SSU rRNA*	*bg*	*tpi*^c^	*gdh*	
Cattle (*Bos taurus*)	47/138 (34.06)	D (1)	D (1)/OM001828		D1 (1)/OM001863	
		E (46)	E1 (1)/OM001829	*E1 *(1)/OM001847	E1 (1)/OM001865	MLG-E1 (1)
				B2 (1), *E1 *(4), **E4 (1)/OM001850**, E6 (1), AI-1/E1 (1)		
			E2 (1)/OM001830	E2 (1)/OM001848		
			E3 (1)/OM001831	**E3 (1)/OM001849**	E1 (1)	MLG-E2 (1)
			E2 (1), E4 (1)/OM001832, E5 (1)/OM001833			
				E5 (1)/OM001851	**E2 (1)/OM001866**	
				*E1 *(1)	E3 (1)/OM001867	
			E2 (2)	*E1* (2)	E1 (2)	MLG-E3 (2)
			E2 (1)	*E1* (1)	E3 (1)	MLG-E4 (1)
			E1 (1)	E6 (1)/OM001852	E4 (1)/OM001868	MLG-E5 (1)
			E3 (1)	*E1* (1)		
				**E7 (1)/OM001853**	E3 (1)	
			E3 (1)	*E1* (1)	E1 (1)	MLG-E6 (1)
			E6 (1)/OM001834	*E1* (1)	E3 (1)	MLG-E7 (1)
			E7 (1)/OM001835	*E1* (1)	E3 (1)	MLG-E8 (1)
Sheep (*Ovis aries*)	63/117 (53.85)	A (2)			AI (1)/OM001856	
			AI (1)/OM001822	AI-1 (1)/OM001840	AI (1)	MLG-AI (1)
		E (61)		E6 (1)		
			E8 (1)/OM001836	E7 (1)	E5 (1)/OM001869	MLG-E9 (1)
			E8 (3)	*E1 *(3)	E6 (3)/OM001870	MLG-E10 (3)
			E3 (1)	E6 (1)	E7 (1)/OM001871	MLG-E11 (1)
			**E9 (1)/OM001837**	E1 (1)	E4 (1)	MLG-E12 (1)
			E8 (1)	E6 (1)	E7 (1)	MLG-E13 (1)
			E3 (1)	*E1 *(1)	E6 (1)	MLG-E14 (1)
					**E8 (1)/OM001872**, E9 (1), **E10 (1)/OM001874**, E11 (1)/OM001875, E13 (1), E14 (1)/OM001878	
			E3 (2)	E6 (2)		
			E8 (1)	*E1* (1)		
			E8 (1)	E7 (1)		
				E6 (1)	E7 (1)	
			E8 (1)	E8 (1)/OM001854	E6 (1)	MLG-E15 (1)
			E3 (1)	*E1 *(1)		
			E10 (1)/OM001838	E8 (1)	E3 (1)	MLG-E16 (1)
			E3 (2)	E1 (2)	E12 (2)/OM001876	MLG-E17 (2)
			E10 (1)		E3 (1)	
				E8 (1), **E9 (1)/OM001855**		
				E8 (1)	E3 (1)	
			E3 (1)	E8 (1)		
			E8 (1)	E1 (1)	E4 (1)	MLG-E18 (1)
			E8 (1)		E13 (1)/OM001877	
			E8 (1)	E1 (1)	E13 (1)	MLG-E19 (1)
			E8 (1)	E2 (1)	E9 (1)/OM001873	MLG-E20 (1)
			E2 (1)	E6 (1)	E4 (1)	MLG-E21 (1)
			E3 (1)	E1 (1)	E3 (1)	MLG-E22 (1)
			AI (1)	AI-1/E6 (1)	AI (1)	
			E8 (1)	AI-1 (1)		
			E8 (1)	E6 (1)	E4 (1)	MLG-E23 (1)
			E3 (1)		E4 (1)	
Pigs (*Sus scrofa domesticus*)	1/59 (1.69)	E (1)				
Reindeer (*Rangifer tarandus*)	9/124 (7.26)	A (7)	AI (1)			
				AI-2 (2)/OM001841		
		E (2)				
Red deer (*Cervus elaphus*)	7/83 (8.43)	A (1), E (6)				
Sika deer (*Cervus nippon*)	5/63 (7.94)	E (5)		AI-1 (1)		
Dogs (*Canis lupus familiaris*)	4/105 (3.81)	C (4)			D2 (1)/OM001864	
Cats (*Felis catus*)	1/17 (5.88)	F (1)				
Raccoon dogs (*Nyctereutes procyonoides*)	1/16 (6.25)	C (1)				
Rabbits (*Oryctolagus cuniculus*)	67/212 (31.60)	B (67)	B2 (3)/OM001825			
				B2 (6)/OM001844		
				B2 (3)	B3 (3)/OM001860	
				B2 (2)	B4 (2)/OM001861	
			B2 (1)		B3 (1)	
			B2 (3)	B2 (3)	B4 (3)	MLG-B2 (3)
			B2 (7)	B2 (7)	B3 (7)	MLG-B3 (7)
			B2 (2)	B3 (2)/OM001845	B3 (2)	MLG-B4 (2)
			**B3 (1)/OM001826**	B3 (1)	B4 (1)	MLG-B5 (1)
			B2 (3)	B3 (3)	B4 (3)	MLG-B6 (3)
			B2 (1)	B4 (1)/OM001846	B3 (1)	MLG-B7 (1)
			B2 (1)	B2 (1)		
			B2 (1)	B3 (1)		
			B4 (1)/OM001827	B2 (1)	B4 (1)	MLG-B8 (1)
			B2 (1)		**B5 (1)/OM001862**	
Brown rats (*Rattus norvegicus*)	9/191 (4.71)	G (9)	**G (1)/OM001839**			
			G (1)		G (1)/OM001879	
					G (2)	
Total	214/1326^b^ (16.14)	A (10), B (67), C (5), D (1), E (121), F (1), G (9)	AI (3), B2–B4 (25), D (1), E1–E10 (42), G (2)	AI (5), B2–B4 (32), E1–E9 (49), AI + E1/E6 (2)	AI (3), B3–B5 (25), D1 (1), D2 (1), E1–E14 (41), G (3)	

The novel sequences obtained in the present study are highlighted in bold

^a^Sika deer and brown rats are wild animals, and the others are domestic animals

^b^Absence of *G. duodenalis* in goats (*Capra hircus*) (*n* = 7), horses (*Equus caballus*) (*n* = 18), arctic foxes (*Vulpes lagopus*) (*n* = 82), blue foxes (*Alopex lagopus*) (*n* = 70) and minks (*Neovison vison*) (*n* = 24) in the present study

^c^At the *tpi* locus, 19 *G. duodenalis*-positive specimens (in italics) were successfully sequenced using general primers and assemblage-specific primers, while the others were only sequenced successfully using assemblage-specific primers

### Processing of fecal specimens

The fecal specimens of herbivores needed to be processed by sieving the crude fiber and impurities in the specimens and concentrating for 10 min at 1500*g*. The fecal specimens of the other animals and the diarrheal patients were simply washed twice with distilled water by centrifugation to concentrate the specimens at the bottom of the centrifuge tube.

### DNA extraction

Genomic DNA of *G. duodenalis* was extracted from approximately 180–200 mg of each washed fecal pellet using a QIAamp DNA Stool Mini Kit (Qiagen, Hilden, Germany). The procedures and reagents used in the present study were provided by the manufacturer. DNA was finally eluted in 200 μl of AE elution buffer and stored at −20 °C until polymerase chain reaction (PCR) analysis.

### PCR amplification

All DNA specimens were screened for the presence of *G. duodenalis* by nested PCR amplification of an approximately 290 base pairs (bp) of the *SSU rRNA* gene, and were identified to the assemblage level as previously designed by Appelbee et al. [30]. All *G. duodenalis*-positive specimens at the *SSU rRNA* locus were further analyzed and identified to the sub-assemblage level by nested PCR amplifications of the *bg*, *tpi*, and *gdh* genes. The fragments of the *bg* (~ 510 bp), *tpi* (~ 530 bp), and *gdh* (~ 530 bp) genes were amplified as previously designed by Lalle et al., Sulaiman et al., and Caccio et al., respectively [31–33]. In addition, to detect more mixed infections of different assemblages in the same specimens, specific nested PCRs were performed to amplify approximately 330 bp (assemblage A), 400 bp (assemblage B), and 390 bp (assemblage E) of the *tpi* gene as previously designed by Geurden et al. and Levecke et al. [34, 35]. DNA extracted from axenic cultures of a human-derived *G. duodenalis* assemblage A isolate was used as positive controls in PCR tests to amplify the *SSU rRNA*, *bg*, *tpi* (general primers and assemblage A-specific primers), and *gdh* genes. DNA of assemblages B and E from a human and a sheep was used as positive controls in assemblage B/E-specific PCR tests to amplify the *tpi* gene, respectively. DNase-free water was used as a negative control in each PCR test. All secondary PCR products were separated by 1.5% agarose gel electrophoresis, following staining with GelStrain (TransGen Biotech, Beijing, China), observed, photographed, and recorded on a Gel Doc™ EZ Gel Documentation System (Bio-Rad, USA).

### DNA sequencing and nucleotide sequence analysis

All positive secondary PCR products of the expected size were sequenced by Comate Bioscience Company Limited (Jilin, China) using the respective secondary PCR primers on an ABI PRISM 3730xl DNA Analyzer using the BigDye Terminator v3.1 Cycle Sequencing Kit (Applied Biosystems, Carlsbad, CA, USA). The nucleotide sequence accuracy was confirmed by two-directional sequencing and by sequencing two more new PCR products if necessary for some DNA specimens in which novel nucleotide sequences were acquired. Nucleotide sequences obtained in the present study were aligned and analyzed with each other and reference sequences of each locus downloaded from GenBank using the Basic Local Alignment Search Tool (BLAST) (http://www.ncbi.nlm.nih.gov/blast/) and the program Clustal X 1.83 (http://www.clustal.org/) to determine the assemblages and sub-assemblages of *G. duodenalis*.

All the specimens successfully amplified and sequenced at the three loci (*bg*, *tpi*, and *gdh*) were included in the multilocus genotyping analysis. Nucleotide sequences of each isolate at the three analyzed loci were concatenated (*bg*-*tpi*-*gdh*) to form one MLG.

The representative nucleotide sequences obtained in the present study were deposited in the GenBank database under the following accession numbers: human-derived *G. duodenalis* isolates—OM001823 and OM001824 (*bg*), OM001842 and OM001843 (*tpi*), and OM001857 to OM001859 (*gdh*); animal-derived *G. duodenalis* isolates—OM001822 and OM001825 to OM001839 (*bg*), OM001840, OM001841 and OM001844 to OM001855 (*tpi*), OM001856 and OM001860 to OM001879 (*gdh*).

### Statistical analysis

Fisher’s exact test and Pearson Chi-square (*χ*^2^) tests implemented in SPSS (Statistical Package for the Social Sciences) 19.0. were used to compare differences in prevalence among different age groups. Odds ratios (ORs) with 95% confidence intervals (CIs) were calculated for the occurrence of *G. duodenalis* in humans. Differences were considered statistically significant at a *P*-value of < 0.05.

## Results

### Prevalence and multilocus genotyping of *G. duodenalis* in humans

Sequence analysis of the partial *SSU rRNA* gene detected *G. duodenalis* in 5.81% (24/413) of human fecal specimens and identified assemblages A (*n* = 5) and B (*n* = 19). *Giardia duodenalis* was distributed in three age groups: 9.78% (22/225) in the minors aged 5–17 years, 1.20% (1/83) in the middle-aged adults aged 36–60 years, and 2.08% (1/48) in the older adults aged > 60 years. By *χ*^*2*^ tests, a significant difference in prevalence was observed only between the age groups of 5–17 and 36–60 years (*χ*^*2*^ = 5.27, *P* = 0.02, OR = 8.89, 95% CI 1.18–62.07) (Table 1).

The 24 DNA specimens positive for *G. duodenalis* at the *SSU rRNA* locus were subjected to PCR amplifications of the *bg*, *tpi*, and *gdh* genes, and three, two (specific PCRs), and three specimens were successfully amplified and subtyped, respectively. All DNA specimens did not have successful PCR amplifications at the *tpi* locus using general primers. By sequence analysis, sub-assemblages AII (*n* = 2) and B1 (*n* = 1) were identified at the *bg* locus, while sub-assemblages AII (*n* = 1) and B1 (*n* = 1) at the *tpi* locus, and sub-assemblages AII (*n* = 1), B1 (*n* = 1) and B2 (*n* = 1) at the *gdh* locus. One *gdh* nucleotide sequence (B1) was not described previously but had 100% similarity with the nucleotide sequences from a human (MG736274), a rabbit (KP635094), and a monkey (MK952603) at the amino acid level (Additional file 1: Tables S1–S3).

Among the 24 *G. duodenalis*-positive specimens from humans, only two were successfully subtyped at the *bg*, *tpi*, and *gdh* loci. Based on concatenated nucleotide sequences of 486 bp (*bg*), 336 bp (*tpi* for A) or 384 bp (*tpi* for B), and 498 bp (*gdh*), two MLGs (MLG-AII and MLG-B1) were produced (Table 1). The MLG-AII isolate had the same nucleotide sequence as a cat-derived isolate (AY072723-U57897-EF685688). The MLG-B1 isolate was not identical to the animal-derived isolates at the nucleotide level, but had 100% homology at the amino acid level with those isolates from a chinchilla (KM977641-KM977637-KM977636), a ring-tailed lemur (KJ888974-KJ888985-AY178753), and a racehorse (MG736242-KY612223-MN174851).

### Prevalence and multilocus genotyping of *G. duodenalis* in animals

Sequence analysis of the partial *SSU rRNA* gene identified *G. duodenalis* in 16.14% (214/1326) of fecal specimens from 11 mammal species. Sheep (*Ovis aries*) (53.85%, 63/117), cattle (*Bos taurus*) (34.06%, 47/138), and rabbits (*Oryctolagus cuniculus*) (31.60%, 67/212) had much higher prevalence than the other animals—red deer (*Cervus elaphus*) (8.43%, 7/83), sika deer (*Cervus nippon*) (7.94%, 5/63), reindeer (*Rangifer tarandus*) (7.26%, 9/124), raccoon dogs (*Nyctereutes procyonoides*) (6.25%, 1/16), cats (*Felis catus*) (5.88%, 1/17), brown rats (*Rattus norvegicus*) (4.71%, 9/191), dogs (*Canis lupus familiaris*) (3.81%, 4/105), and pigs (*Sus scrofa* domesticus) (1.69%, 1/59). Assemblages A to G were identified in the animals. The host distribution of the different assemblages is shown in Table 2.

All 214 DNA specimens positive for *G. duodenalis* at the *SSU rRNA* locus were subjected to PCR amplifications of the *bg*, *tpi*, and *gdh* genes, and 73, 88, and 74 DNA specimens were successfully amplified and subtyped, respectively. Analysis of 73 *bg* gene sequences identified sub-assemblage AI (*n* = 3), B2–B4 (*n* = 25), D (*n* = 1), E1–E10 (*n* = 42), and G (*n* = 2). The two *bg* gene sequences of assemblage G were identical to each other. At the *tpi* locus, 19 *G. duodenalis*-positive specimens were successfully sequenced using general primers, and all of them belonged to assemblage E. Eighty-eight *G. duodenalis*-positive specimens were successfully sequenced using assemblage-specific primers, and were identified as sub-assemblages AI-1 and AI-2 (*n* = 7), B2–B4 (*n* = 32), and E1–E9 (*n* = 51), which covered the above 19 positive specimens (Table 2). Two giardiasis cases of mixed infection with assemblages A and E were detected in one cow and one sheep (Table 3). At the *gdh* locus, 74 nucleotide sequences were obtained, belonging to sub-assemblages AI (*n* = 3), B3–B5 (*n* = 25), D1 and D2 (*n* = 2), E1–E14 (*n* = 41), and G (*n* = 3). The three *gdh* gene sequences of assemblage G were identical to each other (Table 2). Among the 51 representative nucleotide sequences obtained in animals in the present study, 11 and four were not described previously at the nucleotide and amino acid levels (B3, E9, and G versus none at the *bg* locus; E3, E4, E7, and E9 versus E3, E4, and E9 at the *tpi* locus; B5, E2, E8, and E10 versus E8 at the *gdh* locus) (Additional file 1: Tables S1–S3).

**Table 3:** Host distribution of mixed infection of *G. duodenalis* assemblages and sub-assemblages

Host/*n*	Assemblage	Sub-assemblage		
	*SSU rRNA*	*bg*	*tpi*	*gdh*
Cattle (*Bos taurus*)/2	E		AI/E1	
	E		B2	
Sheep (*Ovis aries*)/2	E	AI	AI/E6	AI
	E	E8	AI	
Sika deer (*Cervus nippon*)/1	E		AI	
Dog (*Canis lupus familiaris*)/1	C			D2

Among the 214 *G. duodenalis*-positive specimens from animals, 47 were successfully subtyped at the *bg*, *tpi*, and *gdh* loci. One specimen of mixed infection of assemblages A and E in a sheep was excluded in the multilocus genotyping analysis (Table 3). By analyzing the concatenated nucleotide sequences of 46 specimens (*bg*: 486 bp; *tpi*: 336 bp, 384 bp, and 366 bp for A, B and E, respectively; *gdh*: 498 bp), 31 MLGs were obtained: MLG-AI (*n* = 1), MLG-B2 to MLG-B8 (*n* = 18) and MLG-E1 to MLG-E23 (*n* = 27) (Table 2). Homology analysis suggested that eight MLGs (MLG-AI, MLG-E1, MLG-E3, MLG-E4, MLG-E6, MLG-E8, MLG-E20, and MLG-E22) were reported previously, while the other 23 MLGs were novel. Among them, the MLG-AI isolate from one sheep had 99.84% and 100% similarity with a human-derived isolate (AY655702-EU041756-AB159795) at the nucleotide and amino acid levels, respectively.

### Mixed infection of *G. duodenalis* assemblages

A total of six cases of mixed infection of different assemblages were identified, including B (at the *tpi* locus) + E (at the *SSU rRNA* locus) in one cow, A (at the *tpi* locus) + E (at the *SSU rRNA* and *bg* loci) in one sheep, A (at the *tpi* locus) + E (at the *SSU rRNA* locus) in one sika deer, and C (at the *SSU rRNA* locus) + D (at the *gdh* locus) in one dog, as well as A + E in one cow and one sheep by assemblage-specific nested PCR assays targeting the *tpi* gene (Table 3).

## Discussion

Diarrhea is a common symptom of many conditions. Enteric infections are considered to be the most frequent cause of diarrhea. Investigation of intestinal pathogens will be helpful in developing better and more reasonable treatments for diarrheal diseases. Giardiasis is estimated to account for 0.43–78.8% of all human diarrheal cases worldwide [36]*. Giardia duodenalis* infection is reported to contribute substantially to the 2.5 million annual deaths from diarrheal disease [37]. In our epidemiological investigation of *G. duodenalis* conducted in northeastern China’s Heilongjiang Province, 5.81% of diarrheal patients were infected with *G. duodenalis*. Therefore, early diagnosis and treatment of giardiasis is necessary to decrease the risk of severe clinical consequences of persistent diarrhea.

In the investigated diarrheal patients, a prevalence peak was observed in the 5–17 age group (9.78%). A similar age pattern of *G. duodenalis* infection has been reported in other studies. In Uganda, a significant difference in the prevalence of *G. duodenalis* was reported between individuals 15 years or younger (53.2%) and individuals 16–75 years (22.2%) [38]. In China, children < 15 years of age were the most frequently affected, with the prevalence peak occurring in those aged 5–10 years [25]. The above age distribution characterization of *G. duodenalis* infection could be attributed to poor hygiene and play habits of this age group. All 24 diarrheal patients with giardiasis in the present study claimed no experience of direct contact with animals. However, previous studies have considered that close contact with animals was one of the possible risk factors related to *G. duodenalis* infection. Possible zoonotic transmission has been observed between humans and hoofed animals (cattle, pigs, and horses) in Brazil, Egypt, Canada, Vietnam, and India [15, 16, 20, 39, 40]. *Giardia duodenalis* has a wide animal host range, and it has been identified in various mammal species. In the present study, *G. duodenalis* was found in 16.14% of investigated animals, and was identified for the first time in pigs, reindeer, red deer, sika deer, and brown rats in Heilongjiang Province of China, defining the host distribution of *G. duodenalis* across this region. In the present study, we only identified *G. duodenalis* with zoonotic potential in brown rats, and not *Giardia muris*, which is specific mainly to rodents belonging to the family Muridae [41].

By sequence analysis of the *SSU rRNA* gene, assemblage B (79.2%, 19/24) was observed to be more prevalent than assemblage A (20.8%, 5/24) in diarrheal patients. As the major assemblages A and B causing human giardiasis, assemblage B was responsible for more infections (4083 cases) than assemblage A (2833 cases), based on molecular analysis of 7211 specimens of *G. duodenalis* from two review articles [23, 42]. In the present study, the genotyping results of the *SSU rRNA* gene revealed assemblage constituents of *G. duodenalis* in different animal species: D and E in cattle; A and E in sheep, reindeer, and red deer; E in pigs and sika deer; C in dogs and raccoon dogs; F in cats; B in rabbits; G in brown rats. Because 95.79% (205/214) of animal-derived isolates identified as assemblages A to F are pathogenic for humans, the animals infected with *G. duodenalis* might pose a risk of zoonotic transmission of human giardiasis, and were of public health importance. The identification of assemblage D in cattle was unexpected. Assemblage D is mainly found in canine animals and occasionally seen in other animals, including cattle. To date, only two studies have reported assemblage D in cattle in China and the UK [43, 44]. Based on current data, it was unclear whether assemblage D was a natural infection or mechanical transport in cattle. We were only able to conclude that *G. duodenalis* cysts of assemblage D may have been successfully transmitted to cattle through contaminated water or food.

Sequence analysis of the *bg*, *tpi*, and *gdh* genes exhibited genetic heterogeneity within assemblages A, B, and E in the investigated areas. Two sub-assemblages were identified with AI in animals and AII in humans. To date, identification of the genetic variations in nucleotide sequences within assemblage A at the *bg*, *tpi*, and *gdh* loci has led to the designation of sub-assemblages AI, AII and AIII. They all appear to have different host specificity: AI commonly found in animals, AII largely seen in humans, and AIII mainly detected in wild mammals and rarely in companion animals, cattle, and red deer [45]. By homology analysis, the vast majority (79.31%, 46/58) of nucleotide sequences obtained in the present study were described previously, with 85.71% (6/7) for humans and 78.43% (40/51) for animals. Among them, the vast majority of nucleotide sequences of the human-derived assemblages A and B were identical to those from animals at each of the three analyzed loci, suggesting the possibility of zoonotic transmission in the investigated areas. Meanwhile, some nucleotide sequences of the animal-derived assemblages A, B, and E had been reported in human-derived *G. duodenalis* isolates, suggesting that concerns about the zoonotic transmission of *G. duodenalis* should be directed toward these animals, especially cattle, sheep, reindeer, sika deer, and rabbits infected with zoonotic assemblages A and B. The multilocus genotyping analysis further supported the above presumption of zoonotic transmission of assemblages A and B in the investigated areas. No identical MLGs were found between humans and animals in the present study. Thus, the epidemiological role of animals as reservoir hosts in the transmission of *G. duodenalis* to humans needs to be assessed in the future. For assemblage E, commonly found in hoofed mammals, no identical MLGs were found in cattle and sheep, reflecting limited cross-species transmission of *G. duodenalis* occurring between animals living on the same farms or grazing in the same pastures.

## Conclusion

The present study describes the prevalence and multilocus genetic characterization of *G. duodenalis* in patients with diarrhea and animals in northeastern China’s Heilongjiang Province. The finding of *G. duodenalis* in diarrheal patients enhances awareness regarding the detection of *G. duodenalis* in clinical practice and emphasizes the importance of health education in local inhabitants, especially in the age group of 5–17 years. Seven assemblages (A to G) and 33 MLGs were obtained—A and B (two MLGs) in humans and A to G (31 MLGs) in animals—revealing genetic heterogeneity of *G. duodenalis* in the investigated areas. Assessment of possible zoonotic transmission of *G. duodenalis* was based on homology data mostly at the single locus level and rarely at the multilocus level, and no identical MLGs were observed between the investigated humans and animals. The precise epidemiological role that animals play in the transmission of human giardiasis needs to be assessed by further large-scale molecular epidemiological investigations of local humans and animals.

## Supplementary Information

**Additional file 1: Table S1.** Homology analysis of the *bg* gene of *G. duodenalis* isolates at the nucleotide and amino acid levels. **Table S2.** Homology analysis of the *tpi* gene of *G. duodenalis* isolates at the nucleotide and amino acid levels. **Table S3.** Homology analysis of the *gdh* gene of *G. duodenalis* isolates at the nucleotide and amino acid levels.

## Data Availability

The representative nucleotide sequences obtained in the present study were deposited in the GenBank database under the following Accession Numbers: human-derived *G. duodenalis* isolates—OM001823 and OM001824 (*bg*), OM001842 and OM001843 (*tpi*), and OM001857 to OM001859 (*gdh*); animal-derived *G. duodenalis* isolates—OM001822 and OM001825 to OM001839 (*bg*), OM001840, OM001841 and OM001844 to OM001855 (*tpi*), OM001856 and OM001860 to OM001879 (*gdh*).

## Abbreviations

*SSU rRNA*: Small subunit ribosomal RNA
*bg*: β-Giardin
*tpi*: Triosephosphate isomerase
*gdh*: Glutamate dehydrogenase
PCR: Polymerase chain reaction
BLAST: Basic Local Alignment Search Tool
MLG(s): Multilocus genotype(s)
