# Supplementary material for: Giardia duodenalis in patients with diarrhea and various animals in northeastern China: prevalence and multilocus genetic characterization
Source: Parasit Vectors. 2022 May 11;15:165. doi: 10.1186/s13071-022-05269-9 (PMC9097065; doi:10.1186/s13071-022-05269-9)
Supplement: Supplementary file 1 — Additional file 1: Table S1. Homology analysis of the bg gene of G. duodenalis isolates at the nucleotide and amino acid levels. Table S2. Homology analysis of the tpi gene of G. duodenalis isolates at the nucleotide and amino acid levels. Table S3. Homology analysis of the gdh gene of G. duodenalis isolates at the nucleotide and amino acid levels. [file 13071_2022_5269_MOESM1_ESM.doc]

Additional file 1: Table S1 Homology analysis of the *bg* gene of *G. duodenalis* isolates at the nucleotide and amino acid levels

| Assemblage | Sub-assemblage (Host/no.) | Accession no. ^a^ | Accession no. ^b^ (Host) | Homology | Codon/Amino acid |
| --- | --- | --- | --- | --- | --- |
| A | AI (Sheep/2; Reindeer/1) | OM001822 | MK573339 (Cattle); MK610391 (Sheep); LC437420 (Dog); DQ984131 (Cat); MF497409 (Deer); MF671918 (Chipmunk); KU668890 (Wild boar); KR051224 (Tortoise); JN812308 (Alpaca); AB508814 (Ferret); MK862314 (Camel); MN174848 (Horse) | 100% |  |
|  | AII (Human/2) | OM001823 | MN844143 (Human); MK452860 (Cattle); MK452883 (Sheep) | 100% |  |
| B | B1 (Human/1) | OM001824 | MK982544 (Human); MW727286 (Cat) | 100% |  |
|  | B2 (Rabbit/23) | OM001825 | KM190805 (Human); KC960631 (Rabbit); KJ888980 (Monkey); JX972182 (Horse) | 100% |  |
|  | B3 (Rabbit/1) | OM001826 | KM190805 (Human); KC960631 (Rabbit); KJ888980 (Monkey); JX972182 (Horse) | 99.80% | GA(G→A)/E |
|  | B4 (Rabbit/1) | OM001827 | MK962815 (Human); JN416545 (Dog); MK952608 (Monkey); MF169196 (Horse) | 100% |  |
| D | D (Cattle/1) | OM001828 | KF923019 (Dog) | 100% |  |
| E | E1 (Cattle/2) | OM001829 | MK982550 (Cattle); EU642901 (Goat); MG921621 (Sika deer); KJ917614 (Monkey) | 100% |  |
|  | E2 (Cattle/5; Sheep/1) | OM001830 | MN276322 (Cattle); MK610389 (Sheep); MG921620 (Sika deer); MN174850 (Horse) | 100% |  |
|  | E3 (Cattle/3; Sheep/10) | OM001831 | MH079430 (Cattle); MK610388 (Sheep); MK862313 (Horse) | 100% |  |
|  | E4 (Cattle/1) | OM001832 | MF671886 (Cattle); KJ917613 (Monkey) | 100% |  |
|  | E5 (Cattle/1) | OM001833 | KC960641 (Cattle); DQ116616 (Sheep) | 100% |  |
|  | E6 (Cattle/1) | OM001834 | KC960642 (Cattle) | 100% |  |
|  | E7 (Cattle/1) | OM001835 | MN276323 (Cattle); KP635113 (Sheep); MK862310 (Camel) | 100% |  |
|  | E8 (Sheep/14) | OM001836 | MK573338 (Cattle); MK610387 (Sheep) | 100% |  |
|  | E9 (Sheep/1) | OM001837 | MK573338 (Cattle); MK610387 (Sheep) | 99.80% | AT(C→T)/I |
|  | E10 (Sheep/2) | OM001838 | MT713332 (Cattle); MK442902 (Sheep); KP635098 (Rabbit) | 100% |  |
| G | G (Brown rat*/*2) | OM001839 | MT114176 (Rat) | 99.80% | GG(A→ C)/G |

^a^ Accession no. indicating the nucleotide sequences obtained in this study.

^b^ Accession no. indicating the published nucleotide sequences, which have the largest similarity with the nucleotide sequences obtained in this study.

Additional file 1: Table S2 Homology analysis of the *tpi* gene of *G. duodenalis* isolates at the nucleotide and amino acid levels

| Assemblage | Sub-assemblage (Host/no.) | Accession no. ^a^ | Accession no. ^b^ (Host) | Homology | Codon/Amino acid |
| --- | --- | --- | --- | --- | --- |
| A | AI-1 (Cattle/1; Sheep/3; Sika deer/1) | OM001840 | KY359206 (Human); MK639171 (Sheep); MK452889 (Cattle); MN174857 (Horse); MF497412 (Deer); MF671916 (Chipmunk); KT258020 (Dog); KU531718 (Wombat); KU531710 (Kangaroo); KU531708 (Rabbit); KR051229 (Tortoise); KM926546 (Foal); KJ888991 (Monkey); KF843906 (Chinchilla) | 100% |  |
|  | AI-2 (Reindeer/2) | OM001841 | GQ329677 (Human); MN704937 (Donkey); MN815125 (Cattle); MN174856 (Horse); LC437479 (Dog); MK327163 (Goat); LC341572 (Cat); KM926528 (Foal); JX845436 (Alpaca); MT319052 (Camel) | 100% |  |
|  | AII (Human/1) | OM001842 | MN844148 (Human); MT747424 (Cat) | 100% |  |
| B | B1 (Human/1) | OM001843 | MK982493 (Human); MF459680 (Cattle); MF095053 (Goat); MH644772 (Pig); LC437486 (Dog); LC341576 (Cat); MH475909 (Rabbit); MK909132 (Monkey); KY304079 (Fox); KM190822 (Beaver); KM977637 (Chinchilla); KF843916 (Ferret); GU182372 (Seal); EU518571 (Dolphin); AY228638 (Muskrat) | 100% |  |
|  | B2 (Cattle/1; Rabbit/23) | OM001844 | JX994246 (Human); KM067095 (Cattle); JQ928712 (Sheep); KT372238 (Rabbit) | 100% |  |
|  | B3 (Rabbit/7) | OM001845 | KT124827 (Human); KP635092 (Rabbit) | 100% |  |
|  | B4 (Rabbit/1) | OM001846 | MG637047 (Rabbit) | 100% |  |
| E | E1 (Cattle/14; Sheep/12) | OM001847 | KU378634 (Human); MH230888 (Cattle); MK639173 (Sheep); KJ668132 (Pig); KJ917622 (Monkey); MT319057 (Camel) | 100% |  |
|  | E2 (Cattle/1; Sheep/1) | OM001848 | MN419030 (Cattle); MK442913 (Sheep); MK313802 (Pig); MT319054 (Camel) | 100% |  |
|  | E3 (Cattle/1) | OM001849 | KU378634 (Human); MH230888 (Cattle); MK639173 (Sheep); KJ668132 (Pig); KJ917622 (Monkey); MT319057 (Camel) | 99.73% | A(A→C)G/(K→T) |
|  | E4 (Cattle/1) | OM001850 | KU378634 (Human); MH230888 (Cattle); MK639173 (Sheep); KJ668132 (Pig); KJ917622 (Monkey); MT319057 (Camel) | 99.73% | AT(G→A)/(M→I) |
|  | E5 (Cattle/1) | OM001851 | MG988438 (Cattle); GQ444457 (Sheep) | 100% |  |
|  | E6 (Cattle/2; Sheep/9) | OM001852 | MH158499 (Cattle); MK442915 (Sheep); MT319058 (Camel) | 100% |  |
|  | E7 (Cattle/1; Sheep/2) | OM001853 | MH158499 (Cattle); MK442915 (Sheep); MT319058 (Camel) | 99.73% | GC(A→G)/A |
|  | E8 (Sheep/5) | OM001854 | MK473862 (Cattle); JF792420 (Sheep) | 100% |  |
|  | E9 (Sheep/1) | OM001855 | MH158499 (Cattle); MK442915 (Sheep); MT319058 (Camel) | 99.73% | (G→A)CC/(A→T) |

^a^ Accession no. indicating the nucleotide sequences obtained in this study.

^b^ Accession no. indicating the published nucleotide sequences, which have the largest similarity with the nucleotide sequences obtained in this study.

Additional file 1: Table S3 Homology analysis of the *gdh* gene of *G. duodenalis* isolates at the nucleotide and amino acid levels

| Assemblage | Sub-assemblage (Host/no.) | Accession no. ^a^ | Accession no. ^b^ (Host) | Homology | Codon/Amino acid |
| --- | --- | --- | --- | --- | --- |
| A | AI (Sheep/3) | OM001856 | MK645799 (Sheep); MW138913 (Dog); MF671911 (Chipmunk); MT319049 (Camel); MN174853 (Horse); EF507600 (Cat); MH051905 (Reindeer) | 100% |  |
|  | AII (Human/1) | OM001857 | MK962825 (Human); KY432844 (Cattle); MN480451 (Giraffe) | 100% |  |
| B | B1 (Human/1) | OM001858 | MG736274 (Human); KP635094 (Rabbit); MK952603 (Monkey) | 99.80% | AA(C→T)/N |
|  | B2 (Human/1) | OM001859 | MK982472 (Human); KM977636 (Chinchilla) | 100% |  |
|  | B3 (Rabbit/14) | OM001860 | MG736274 (Human); KP635094 (Rabbit); MK952603 (Monkey) | 100% |  |
|  | B4 (Rabbit/10) | OM001861 | EU834844 (Human); KC960646 (Rabbit); KM977635 (Chinchilla) | 100% |  |
|  | B5 (Rabbit/1) | OM001862 | MH311013 (Human); KP635094 (Rabbit); MK952603 (Monkey) | 99.80% | CT(C→T)/L |
| D | D1 (Cattle/1) | OM001863 | JN587398 (Dog) | 100% |  |
|  | D2 (Dog/1) | OM001864 | KR855636 (Dog) | 100% |  |
| E | E1 (Cattle/5) | OM001865 | KY655480 (Human); MK573340 (Cattle); KJ668138 (Pig); KJ917618 (Monkey) | 100% |  |
|  | E2 (Cattle/1) | OM001866 | KY432839 (Cattle) | 99.80% | CG(C→T)/R |
|  | E3 (Cattle/5; Sheep/4) | OM001867 | MK982483 (Human); MK573341 (Cattle); MK645797 (Sheep) | 100% |  |
|  | E4 (Cattle/1; Sheep/5) | OM001868 | KY769097 (Cattle); MK645789 (Sheep) | 100% |  |
|  | E5 (Sheep/1) | OM001869 | KT369780 (Cattle) | 100% |  |
|  | E6 (Sheep/5) | OM001870 | MK442907(Sheep) | 100% |  |
|  | E7 (Sheep/3) | OM001871 | KP635095 (Rabbit) | 100% |  |
|  | E8 (Sheep/1) | OM001872 | KY432840 (Cattle); MK442909 (Sheep) | 99.80% | G(T→C)C/(V→A) |
|  | E9 (Sheep/2) | OM001873 | MF671895 (Cattle); MK645786 (Sheep); MN047216 (Camel) | 100% |  |
|  | E10 (Sheep/1) | OM001874 | MK442907 (Sheep) | 99.80% | GT(C→T)/V |
|  | E11 (Sheep/1) | OM001875 | KY432840 (Cattle); MK442909 (Sheep) | 100% |  |
|  | E12 (Sheep/2) | OM001876 | KP334146 (Cattle); MT319050 (Camel) | 100% |  |
|  | E13 (Sheep/3) | OM001877 | KY432864 (Sheep) | 100% |  |
|  | E14 (Sheep/1) | OM001878 | KY769098 (Cattle); MK645788 (Sheep) | 100% |  |
| G | G (Brown rat/3) | OM001879 | MT114177 (Rat) | 100% |  |

^a^ Accession no. indicating the nucleotide sequences obtained in this study.

^b^ Accession no. indicating the published nucleotide sequences, which have the largest similarity with the nucleotide sequences obtained in this study.
